# Characterization of pulmonary bacterial composition in community-acquired pneumonia depending on sampling methods

**DOI:** 10.3389/fmicb.2026.1938648

**Published:** 2026-10-09

**Authors:** Luis Cabanes López, Miguel Carda-Diéguez, Alejandro Mira, Eva Martínez-Moragón

**Affiliations:** 1 University Hospital Pulmonology Service La Ribera, Alzira, Spain; 2 Genomics and Health Department, FISABIO Foundation, Valencia, Spain; 3 University Hospital Pulmonology Service Dr. Peset, Valencia, Spain; 4 CIBER Center for Epidemiology and Public Health, Madrid, Spain

**Keywords:** 16S rRNA sequencing, community-acquired pneumonia, diagnostic methodology, pulmonary microbiome, *Ralstonia*, respiratory infection

## Abstract

**Introduction:**

Community-acquired pneumonia (CAP) remains a major global health concern due to its high incidence, morbidity, mortality, and economic impact. Although *Streptococcus pneumoniae* is the primary bacterial cause, diverse bacterial and viral pathogens contribute to CAP etiology. Conventional microbiological methods provide limited characterization of the pulmonary microbiome, resulting in low diagnostic yield and frequent empirical antibiotic use. This pilot study aimed to characterize the pulmonary microbiome in adult CAP patients using high-throughput sequencing and to determine whether commonly used non-invasive samples accurately represent the lower respiratory tract.

**Methods:**

Pulmonary samples were obtained using invasive procedures (bronchoalveolar lavage, bronchial aspirate, bronchial brushing, and biopsy) and non-invasive methods (sputum, nasopharyngeal aspirate). Classical microbiological testing identified pathogens in 24.1% of cases. DNA extracted was subjected to 16S rRNA gene amplification and sequencing, and microbial composition and diversity were compared across sampling types.

**Results:**

Microbiome profiles differed significantly by sampling methodology. Non-invasive samples displayed high variability and limited correspondence with lower airway microbiota. In contrast, invasive samples demonstrated greater consistency and appeared more representative of the lower respiratory tract. The genus *Ralstonia* was significantly more abundant in invasive samples (*p* < 0.05), warranting further investigation of its potential biological and clinical relevance.

**Conclusions:**

16S rRNA gene sequencing provides comprehensive detection of pulmonary microorganisms and offers added value in the microbiological diagnosis of CAP. The findings highlight the importance of using invasive specimens for accurate microbiome characterization and for detecting bacterial taxa not identified by routine microbiological methods.

## Introduction

Pneumonia, a prevalent and potentially life-threatening respiratory infection, stands as a significant global health concern. This infectious disease targets the lungs, leading to inflammation of the air sacs, and can be caused by various pathogens such as bacteria, viruses, fungi, or parasites (Apisarnthanarak and Mundy, 2005; Prina et al., 2015). Pneumonia's diverse etiology, coupled with its ability to affect individuals across age groups and socioeconomic backgrounds, underscores the importance of a thorough scientific exploration to comprehend its complexities. Following the recent pandemic, timely etiological diagnosis has become even more critical for appropriate clinical management. Community-Acquired Pneumonia (CAP) stands as a distinctive and clinically significant subset within the broader spectrum of respiratory infections. Unlike hospital-acquired or healthcare-associated pneumonia, CAP is characterized by its onset outside of healthcare facilities, affecting individuals in the community (Apisarnthanarak and Mundy, 2005; Bjarnason et al., 2018). *Streptococcus pneumoniae* remains a leading bacterial culprit, while other bacteria (*Mycoplasma pneumoniae, Legionella pneumophila*, and *Staphylococcus aureus*) and viruses such as influenza, respiratory syncytial virus, and human metapneumovirus contribute significantly to the viral etiology (Qu et al., 2015; Cilloniz et al., 2016).

Optimal management of CAP involves a judicious approach to antimicrobial therapy based on the likely causative agent. To this end, non-invasive samples (such as sputum in most cases) are often cultured in microbiology laboratories for pathogen identification (Metlay et al., 2019). Among the non-invasive diagnostic techniques in CAP, detection of antigens in urine for pneumococci and *Legionella* stands out for its high specificity (only 3% false positives) and sensitivity (80%) (Sinclair et al., 2013; Wu and Segal, 2018), as well as Gram staining of sputum with variable sensitivity and specificity due to the challenge of obtaining valid and high-quality samples (57%−95% sensitivity and 48%−87% specificity) (Sinclair et al., 2013). In invasive samples (bronchial aspirate, bronchoalveolar lavage), Gram staining has high specificity (90%). Nevertheless, microbiological diagnosis of CAP remains challenging, achieving successful diagnosis in no more than 60% of cases, often leading to empirical antibiotic use and increased antimicrobial resistance (Vaughn et al., 2024; Menéndez et al., 2020; Pettigrew et al., 2021). Most microorganisms cannot be cultured using standard techniques due to lacking appropriate conditions or methods. In this regard, molecular sequencing techniques have played a crucial role in revealing the association between lung diseases and dysbiosis of the pulmonary microbiome (Wu and Segal, 2018; Li et al., 2024).

The aim of this study was to evaluate high-throughput sequencing in different sample types as a method to enhance the detection of pathogens obtained through traditional techniques based on cultures, urine antigen, and polymerase chain reaction of specific pathogens. We hypothesized that studying microbiomes in the lower respiratory tract could be instrumental for understanding susceptibility to lung infections such as CAP. Therefore, we characterized the pulmonary microbiome of adult patients with CAP through Illumina sequencing and analyzed the differences in bacterial composition associated with the use of non-invasive and invasive methodologies.

## Material and methods

### Study design and period

A prospective observational cohort study was carried out by the Pulmonology Service in the tertiary Hospital from Valencia (Spain) Dr. Peset University Hospital. The study was approved by the ethic committee of the Health Department of Dr. Peset Hospital (code: 76/18). We recruited patients admitted to the Pneumology hospital ward with a diagnosis of CAP between November 2018 and November 2019. Fifteen adult patients were admitted. These patients were more than 18 years old and did not meet the criteria for admission to the Intensive Care Unit based on the scales proposed by the Spanish Society of Pneumology and Thoracic Surgery (Metlay et al., 2019; Menéndez et al., 2020) did not have any serious comorbidity that prevented, conditioned or advised against the study, did not have contraindication of performing a bronchoscopy technique and/or refused informed consent.

## Collection of samples

Patients who met admission criteria with a diagnosis of CAP at the emergency department were transferred to the Pulmonology ward. Once hospitalized, they were given information orally and through written informed consent of the study. Patients could decide how many invasive sampling approaches to authorize, which affected sample size for different sample procedures. Although the inclusion of patients was consecutive, in patients that signed the consent on a non-working day, the techniques were performed on the first working day and in no case there was antibiotic therapy withdrawn. However, the type of antibiotic and the duration of treatment was always recorded in the clinical history (Figure 1).

**Figure 1:**
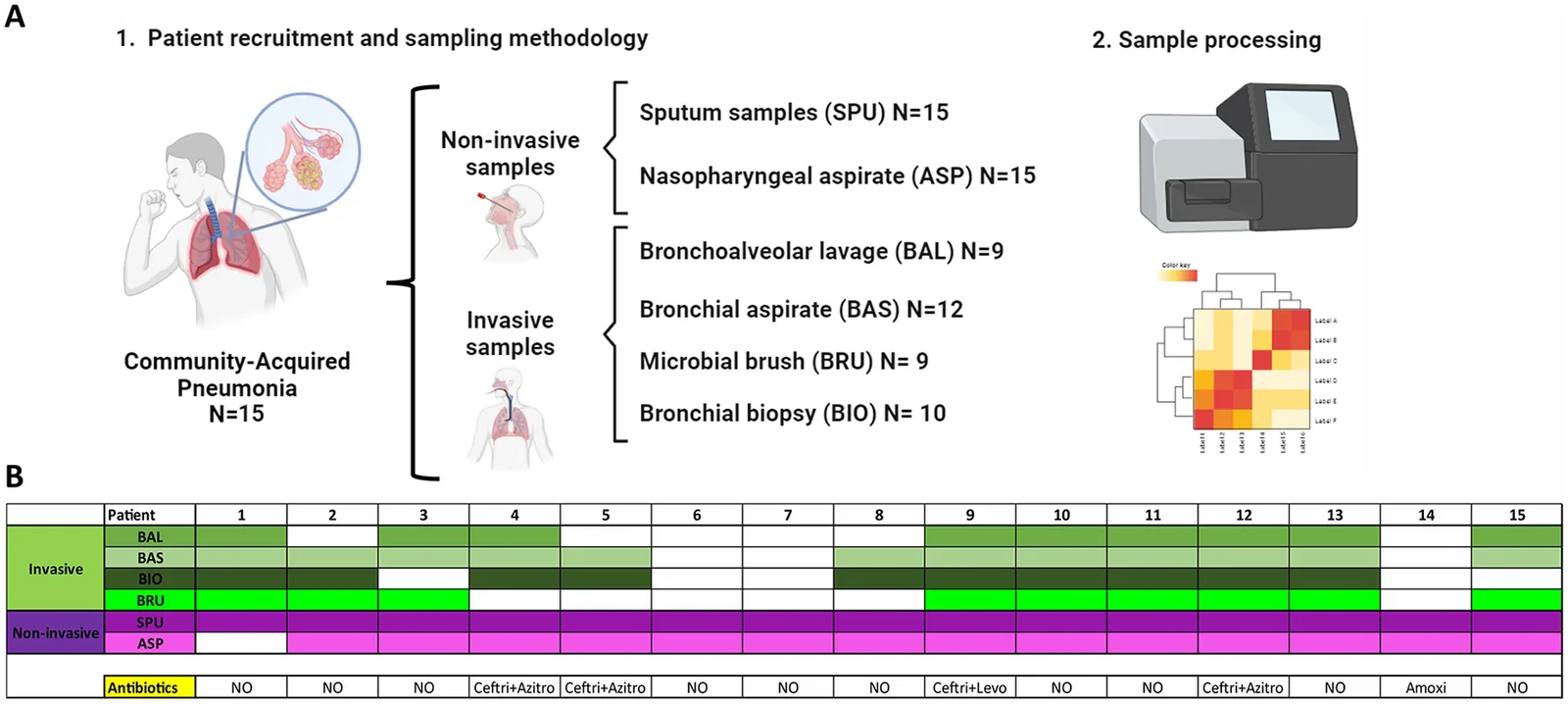
Study design figure. Part **(A)** summarizes the type of samples and how these were analyzed. Fifteen CAP patients were recruited, informed about the study and requested for participation. Six types of sampling methodologies were initially intended for each participant. DNA from each sample was extracted and sequenced using Illumina technology and, finally, analyzed to compare the differences in composition depending on the sampling methodology. In part **(B)**, the samples obtained for each patient and the antibiotics that patients took before admission into the hospital are included in a table. *NO*, no antibiotic was taken; *Ceftri*, Ceftriaxone; *Azitro*, Azithromycin; *Levo*, Levofloxacin; *Amoxi*, Amoxicillin.

Regarding the collection of microbiological samples, 99 samples have been analyzed using high-throughput sequencing techniques. During admission, non-invasive samples were taken: sputum (SPU), and nasopharyngeal aspirate (ASP). Invasive microbiological tests (only collected during admission) were performed using a bronchoscopic technique. Bronchoalveolar lavage (BAL), bronchial aspirate (BAS), microbiological brush (BRU) and bronchial biopsy (BIO) samples were collected.

## Non-invasive samples

The non-invasive samples were collected upon hospital admission, once patients were admitted to the Pulmonology ward. SPU, ASP, blood cultures, and urinary antigen tests were obtained as part of the conventional diagnostic work-up and processed in the clinical laboratory following standardized protocols. Culture-based methods were applied to the corresponding respiratory and blood samples, whereas urinary antigens were assessed using specific antigen-detection assays. Of these, only SPU and ASP samples were further selected for sequencing analysis, enabling a detailed molecular characterization of the respiratory bacterial communities.

## Invasive microbiological samples

The bronchoscopy was carried out by a bronchoscopist pulmonologist together with a nursing assistant and an assistant, in the bronchoscopy room of the Pneumology service; under sedation and patient monitoring, prior explanation of technique and signature of specific informed consent for bronchoscopy. The BAL and BAS were preferably performed on the segment affected by pneumonia. A total of 150 ml of physiological saline was instilled, separated into three syringes of 50 ml each. The first syringe constituted the BAS, while the second and third formed part of the BAL as they were more sterile samples. Regarding the BRU and BIO, we initially performed the BRU, preferably in the pulmonary segment most affected by inflammation. Finally, we carried out the BIO, as it is the invasive test with the highest risk of bleeding among the four. All the samples collected from the patients (invasive and non-invasive) were also processed using the usual culture techniques, in order to be able to make a subsequent comparison of results (Baughman, 2007; Du Rand et al., 2011).

All samples were labeled and stored at −80 °C, until they were transferred to FISABIO Institute (Valencia). To ensure the reliability of the results—particularly in low-biomass samples—strict contamination control measures were implemented. On one hand, after each procedure, bronchoscopes underwent a rigorous standardized reprocessing protocol, including immediate pre-cleaning, leak testing, thorough manual cleaning with dedicated channel brushing, extensive rinsing, and high-level disinfection through immersion in ortho-phthalaldehyde or glutaraldehyde solutions, performed using an automated washer (Medivators ISA Endoscope Reprocessor). Finally, comprehensive drying with filtered medical-grade air was carried out to prevent bacterial proliferation. On the other hand, the sterile saline solution used for bronchoalveolar lavage was sequenced as a negative control in each batch in order to identify and filter out environmental or kit-associated contaminants. Liquid used to transport the samples (KabiPac sodium chloride 9 mg/ml. ref 616003.9) was treated and sequenced as the rest of the samples and used as a negative control to discard potential contaminants.

## DNA extraction and sequencing

DNA was extracted using the MagNa Pure LC DNA Isolation kit II in a MagNa Pure Robot (Roche), following the manufacturer instructions with some modifications (Dzidic et al., 2018). DNA concentration was calculated with the Quant-iT™ PicoGreen^®^ dsDNA Assay Kit and a Qubit™ 3 Fluorometer (Thermo Fisher Scientific, Waltham, MA, USA). The V3-V4 hypervariable region of the 16S rRNA gene was amplified using the universal primer Forward (CCTACGGGNGGCWGCAG) and Reverse (GACTACHVGGGTATCTAATCC) optimized for Illumina sequencing as previously described (Dzidic et al., 2018). Library was constructed using the Metagenomic Sequencing Library Preparation Illumina protocol (Part #15044223 Rev. A). Sequencing was performed at the FISABIO Institute (Valencia, Spain) using an Illumina MiSeq instrument with 2 × 300 bp paired-end. In addition, a negative control was included during library preparation and sequencing to monitor potential contamination introduced during these steps. Sequencing data have been publicly deposited in the SRA database (Bioproject PRJNA859796).

## Bioinformatic analysis of sequencing data

Paired-end reads were processed using DADA2 v1.16 (Callahan et al., 2016) for quality filtering, trimming, denoising, merging of paired reads, and inference of amplicon sequence variants (ASVs). Adapters and primers were removed, reads were end-trimmed in 10-bp windows with quality values < 35, and reads containing ambiguous bases were discarded. Chimeric sequences were removed, and taxonomic assignment of ASVs was performed using the SILVA non-redundant database v138.1 (Quast et al., 2013).

Potential contaminant sequences were identified at the ASV level using the prevalence-based method implemented in the R package “decontam” v1.22.0 (Davis et al., 2018). Five sequenced negative-control libraries, including transport saline and extraction controls, were compared with biological samples using the default contamination threshold of 0.1. ASVs classified as potential contaminants were removed before taxonomic aggregation and downstream analyses.

Richness and alpha-diversity analyses were performed using a separate OTU-based workflow in which sequences were clustered at 97% sequence identity using VSEARCH v2.18.0 (Rognes et al., 2016). Singleton OTUs were retained for these analyses. The resulting OTU abundance table was rarefied to 10,000 reads per sample before calculation of richness and diversity indices.

Downstream statistical analyses were performed in R (R Core Team, 2022). Differential abundance analyses at the genus and species levels were performed using ANCOM-BC2 (Lin and Peddada, 2020) including patient ID as a fixed effect to account for repeated measurements from the same individual. Pairwise comparisons between sampling methods were performed using paired Wilcoxon signed-rank tests and included only patients for whom both sample types being compared were available. Constrained correspondence analysis (CCA) was performed using the “vegan” package to visualize differences in microbial composition among sample types. Multiple-testing correction was performed using the false discovery rate (FDR) method.

## Results

Respiratory samples were obtained using invasive and non-invasive sampling protocols (Figure 1). DNA from samples from each individual was extracted, PCR-amplified for the 16S rRNA gene and sequenced using MiSeq technology. In addition, a total of 62 samples from the 15 patients were subject to analysis using conventional microbiological diagnostic methods. These included bronchoscopic samples (*n* = 11 BAL, *n* = 12 BAS, and *n* = 5 BRU), blood samples (*n* = 7 blood cultures), urine samples (*n* = 14 urinary antigen tests), and upper respiratory tract samples (*n* = 13 SPU). Following analysis using standard routine techniques in the hospital laboratory, the microbiological positivity rate was 24.19% (15/62: three SPU, three urinary antigen tests, three blood cultures, one BAL, five BAS, and zero BRU). This low diagnostic yield obtained through conventional methods is consistent with published evidence indicating that only approximately 30% of CAP cases achieve a definitive microbiological diagnosis using these techniques.

In order to determine if lung-associated microbiota composition depended on whether the sample was obtained using invasive or non-invasive approaches, we compared those groups of samples. Both at the genus and species level, the microbiota presented significant differences when comparing sampling methodologies. Rarefaction curves suggested that most of the diversity was covered for all samples using 10^4^ reads (Figure 2A). Diversity analyses showed a significant (*p* < 0.05) reduction in both Shannon diversity and Chao1 richness indexes when samples were obtained using ASP in comparison to SPU (Figure 2B). This indicates that even between commonly accepted non-invasive samples considerable differences could be detected. In addition, invasive samples also showed significant differences among them in both indexes when compared among them. However, no clear pattern was observed when comparing invasive vs. non-invasive sampling methodologies since BIO (invasive) and SPU (non-invasive) showed the highest diversity among the sample types.

**Figure 2:**
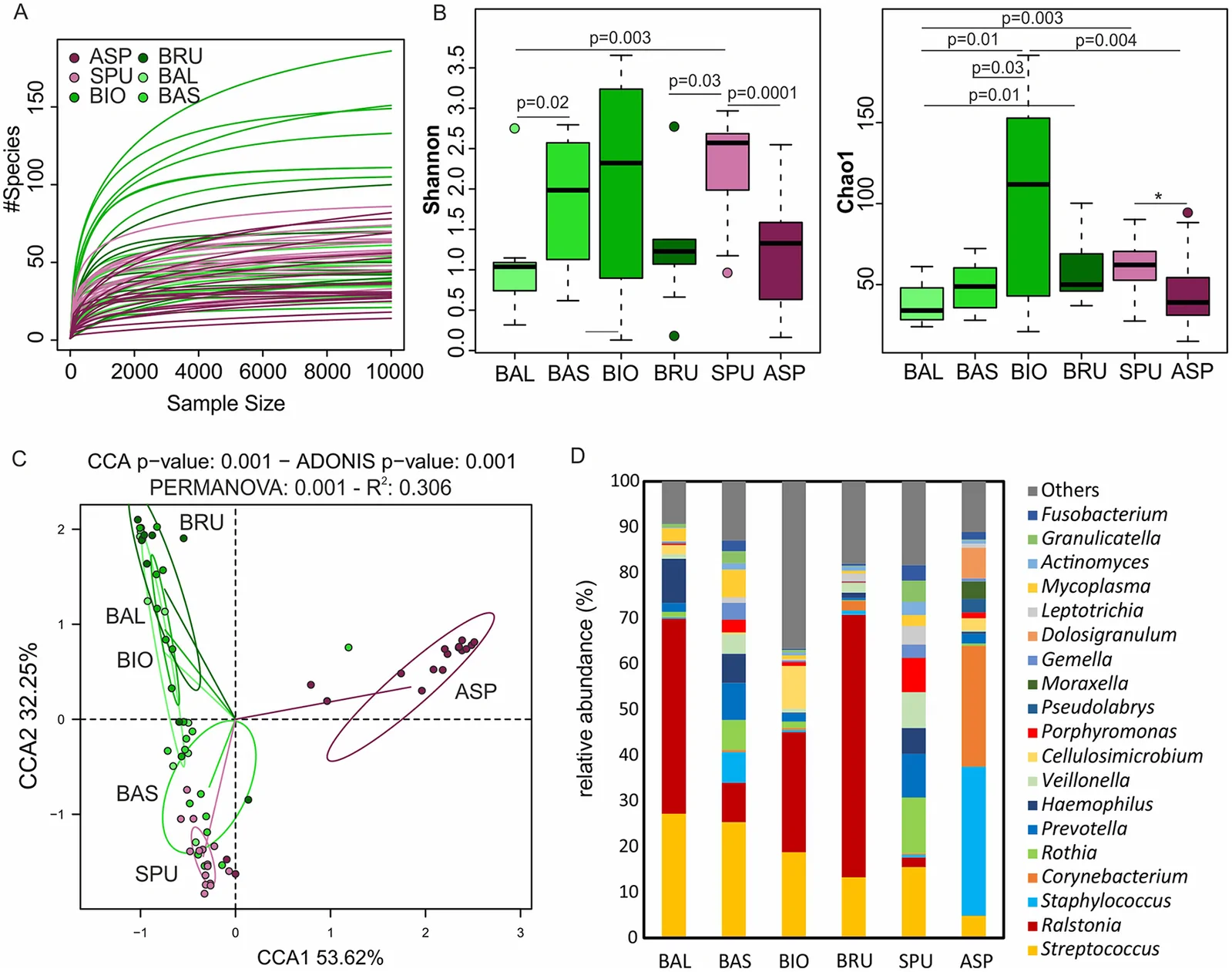
Pulmonary-associated microbial changes. Invasive (green) and non-invasive (purple) samples are differentiated by color. **(A)** Rarefaction curves at the species level using the minimum number of reads annotated at the species level (10,000). **(B)** Shannon (evenness) and Chao1 (richness) alpha diversity indexes. **(C)** Canonical correspondence analyses (CCA) of the microbiota. ADONIS test was used to evaluate the significance of the clustering. **(D)** Top-20 most abundant genera in each group. *p-value < 0.05.

Regarding microbial composition, the CCA plots at the species level showed a significant clustering of samples obtained through invasive protocols, especially in the BAL, BIO, and BRU (Figure 2C). ASP formed a separate cluster from the SPU, while SPU clustered closer to BAS. In fact, when comparing these two sample types, no significant differences were found. Considering that BIO samples represent the microbiota associated with the original infection tissue, BRU and BAL samples showed the highest similarity to BIO (*p* < 0.001). This suggests that BRU and BAL could be considered representative samples of the microbiota detected in biopsies. On the other hand, it is observed that the microbiota found in the nasopharynx (ASP) differs significantly from that found in samples from the lower respiratory tract and in samples such as SPU.

When looking at the composition at the genus level, differences in relative abundance were observed. The abundance of *Streptococcus* was 21% ± 6% in invasive samples and 20% ± 14% in SPU whereas it got reduced up to 4.2% ± 8.8% in ASP. In contrast, *Staphylococcus* (26.4% ± 1.7%) and *Corynebacterium* (23.7% ± 0.3%) showed high relative abundances in ASP (Figure 2D) and were significantly more abundant in these samples than in the other sample types (adjusted *p* < 0.05; Figure 3).”

**Figure 3:**
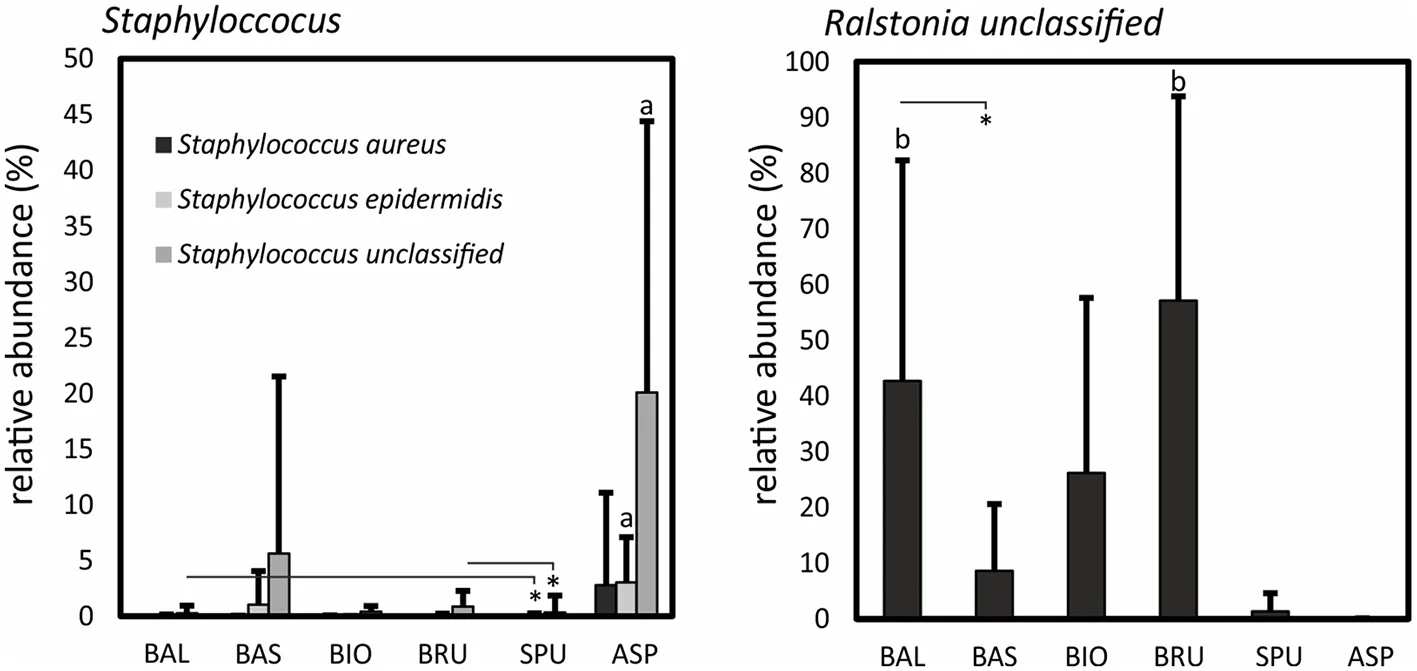
Differences in the abundance of *Staphylococcus* species and unclassified *Ralstonia*. *, adjusted *p*-value < 0.05 between two groups; a, adjusted *p*-value < 0.05 when comparing ASP against any other group; b, adjusted *p*-value < 0.05 when comparing against ASP.

The comparison of non-invasive samples (ASP vs. SPU) resulted in 12 species changing significantly (all of them with >0.1% of relative abundance). Among invasive samples, most of the differences were driven by comparisons involving BAS. In contrast, comparisons restricted to BAL, BIO and BRU identified only one species (BAL vs. BIO; *Bosea unclassified*) with an adjusted *p* < 0.05, supporting the greater similarity among these three sampling methods observed in the CCA. Differences were observed in the abundance of species commonly associated with pneumonia, such as *Streptococcus pneumoniae, Mycoplasma pneumoniae, Legionella pneumophila, Haemophilus influenzae, Staphylococcus aureus*, and *Moraxella* spp. For instance, *S. pneumoniae* was more abundant (not significantly) in invasive samples suggesting that *S. pneumoniae* could be under-represented in commonly used non-invasive samples. Therefore, even though *Streptococcus* as a genus was equally found in invasive and SPU samples, *S. pneumoniae* was not. Similarly, *M. pneumoniae, L. pneumophila*, and *H. influenzae* showed similar profiles but the differences were not significant. Meanwhile, *Staphylococcus aureus* was more abundant in non-invasive samples, in particular in ASP (Figure 3).

Another finding was the higher abundance of *Ralstonia* in invasive samples (BAS and BRU) in comparison to ASP and SPU (adjusted *p* < 0.05; Figure 3). At the species level, these reads remained unclassified within the SILVA database. Given that *Ralstonia* is frequently associated with environmental and reagent contamination, we formally assessed potential contaminants using the prevalence-based method implemented in the R package decontam. Of the 4,532 ASVs evaluated, 51 were classified as potential contaminants at the default threshold of 0.1 and were removed from downstream analyses. Six ASVs were assigned to the genus *Ralstonia*, none of which were classified as contaminants. Moreover, no reads assigned to *Ralstonia* were detected in any of the sequenced negative-control libraries. These findings argue against transport-buffer or extraction-reagent contamination as the source of the *Ralstonia* signal observed in biological samples. Nevertheless, contamination introduced during bronchoscopy cannot be completely excluded in the absence of procedural bronchoscope controls. The bacterial DNA present in these negative controls corresponded to environmental genera frequently associated with contamination from DNA extraction kits or water sources (Olomu et al., 2020; Stinson et al., 2019). Given that some *Ralstonia* species, such as *R. insidiosa*, have been associated with nosocomial infections (Wang et al., 2024; Xi et al., 2024), this finding in the lower respiratory tract warrants further investigation to elucidate its potential clinical role in CAP.

Finally, empirical antibiotic use was considered when analyzing differences in the microbial composition of invasive and non-invasive pulmonary samples. Although the observational design of the study inevitably introduced heterogeneity in both the type and timing of antibiotic administration, we performed a comparative analysis to assess taxonomic shifts. The relative abundance of bacterial species commonly associated with pneumonia—including *S. pneumoniae, M. pneumoniae, L. pneumophila, H. influenzae, S. aureus*, and *Moraxella* spp. (including *Moraxella atalantae*, unclassified *Moraxella*, and *Moraxella nonliquefaciens*)—was affected by previous antibiotic exposure (Supplementary Figure 1). We observed two main patterns. First, some bacteria showed decreased abundance in patients who had received antibiotics, regardless of sample type; these included *S. pneumoniae, Haemophilus* spp., and *L. pneumophila*. Second, other species increased in abundance following antibiotic treatment, including *Staphylococcus* spp. and *M. pneumoniae*, indicating that they were not affected by the antibiotics used. Additionally, unclassified *Ralstonia*, which was present at high levels in several individuals, was not affected by antibiotic exposure. Although these findings suggest that prior antibiotic use modulates the pulmonary microbiome in patients with CAP, the limited sample size and the exploratory nature of the study preclude definitive clinical correlations. These data underscore the importance of interpreting CAP microbiome studies in the context of early empirical treatment, highlighting the need for standardized sampling-to-treatment time windows in future clinical research.

## Discussion

The diagnosis of community-acquired pneumonia (CAP) using conventional microbiological methods remains limited, with only about 30% of cases achieving an etiologic diagnosis after culture and Gram staining (Fong, 2020). In our study, these routinely used methodologies identified pathogens in fewer than one-quarter of cases, underscoring the need for complementary techniques. Sequencing of the 16S rRNA gene has contributed substantially to understanding the microbial ecology of the lower respiratory tract and the disruption of the healthy lung microbiome during disease (Chen et al., 2021; Dickson et al., 2016). Leveraging this technology, we aimed to characterize the lung microbiome in CAP using both invasive and non-invasive samples and to evaluate differences in microbial composition as well as the potential diagnostic utility of these sample types.

Our findings reveal substantial differences between sample types in terms of microbial diversity, composition, and diagnostic yield. Non-invasive samples from the upper respiratory tract, particularly nasopharyngeal aspirates (ASP) and sputum (SPU), demonstrated high variability and limited correspondence with the microbiota detected in lower respiratory tract samples. ASP showed the greatest divergence, suggesting limited correspondence between nasopharyngeal and lower respiratory tract microbial profiles. SPU samples were also heterogeneous, likely reflecting oral microbial contamination during collection, as previously reported (Cabrera-Rubio et al., 2012). These differences may limit the ability of non-invasive respiratory specimens to accurately reflect the microbial composition of the lower respiratory tract.

In contrast, invasive samples demonstrated higher internal consistency and distinct microbial profiles compared with non-invasive samples. Canonical correspondence analysis separated invasive and non-invasive specimens, reflecting the distinct ecological niches they represent. BAL, BRU, and BIO samples clustered closely, consistent with previous studies indicating that these invasive methodologies provide comparable microbiological information (Cabrera-Rubio et al., 2012). Notably, BRU and BAL showed microbial profiles similar to those obtained from bronchial biopsies, suggesting that these sampling approaches may provide comparable information on bacterial community composition. BAS samples showed intermediate behavior, clustering with SPU rather than with other invasive samples, likely due to oropharyngeal contamination during sampling. Overall, BAL and BRU showed the closest correspondence with the microbial profiles detected in biopsy samples, suggesting that they may better reflect lower respiratory tract microbial composition than the non-invasive specimens evaluated in this study. At the genus level, *Streptococcus* predominated in invasive samples, whereas *Staphylococcus*, and *Corynebacterium* were more abundant in non-invasive samples, particularly ASP. These differences highlight the potential for misinterpretation of microbial results when non-invasive sampling is used as a surrogate for lower respiratory tract conditions.

Among the bacterial microorganisms commonly associated with CAP, we detected *Streptococcus pneumoniae, Legionella pneumophila, Haemophilus* spp., *Mycoplasma* spp., and *Staphylococcus aureus*. Invasive specimens also revealed additional bacteria taxa that have been less frequently reported in CAP, highlighting differences in microbial composition between invasive and non-invasive respiratory specimens. *Ralstonia* showed markedly higher abundance in invasive specimens, whereas its abundance was comparatively low in BAS samples, which clustered more closely with SPU samples. This pattern may reflect differences in the contribution of lower respiratory tract and oropharyngeal microbiota across sampling methods and suggests that certain bacterial taxa may be underrepresented in non-invasive specimens. These species-level assignments should, however, be interpreted with caution, as the taxonomic resolution provided by short-read sequencing of the V3–V4 region of the 16S rRNA gene varies among bacterial genera and may not reliably distinguish closely related species, particularly within groups such as *Streptococcus*.

The identification of *Ralstonia* in CAP has been documented recently (Wang et al., 2024), where it appeared as the most abundant genus in BAL samples. The genus has traditionally been associated with nosocomial contamination (Küçükbayrak et al., 2009; Ma et al., 2023; Pan et al., 2011; Zong and Peng, 2011), several observations in our study argue against contamination from the transport buffers or extraction reagents. No reads assigned to *Ralstonia* were detected in any of the sequenced negative-control libraries, and none of the six *Ralstonia*-assigned ASVs were classified as contaminants by the prevalence-based *decontam* analysis. In addition, the bronchoscope underwent a standardized reprocessing protocol after each procedure, including immediate pre-cleaning, leak testing, through manual cleaning with dedicated channel brushing, extensive rinsing, and high-level disinfection by immersion in ortho-phthalaldehyde or glutaraldehyde solutions using an automated washer. Although these measures reduce the likelihood of contamination, they cannot completely exclude contamination introduced during bronchoscopy or sample collection. Moreover, because the study did not include healthy controls and 16S rRNA sequencing does not establish pathogenicity, these findings should not be interpreted as evidence that *Ralstonia* is a causative agent of CAP. Rather, they support further investigation into the potential biological or clinical relevance of *Ralstonia*, particularly in patients with negative results in standard diagnostics.

Antibiotic exposure prior to hospital admission also influenced microbial patterns. Patients who had received antibiotics showed reduced abundance of classical CAP pathogens such as *S. pneumoniae, Haemophilus* spp., and *L*. *pneumophila*, but increased *Staphylococcus* spp. and *M. pneumoniae*. These shifts suggest competitive interactions within the lung microbiome during antibiotic perturbation. While evidence on the rapid induction of lung dysbiosis in humans is limited, findings from cystic fibrosis and animal models indicate that pulmonary microbiota can exhibit significant changes within days of antibiotic therapy (Carmody et al., 2022; Perdijk et al., 2024; Inam et al., 2022). Although our sample size limits definitive conclusions, these observations reinforce the clinical need for early and accurate pathogen detection to guide targeted antimicrobial therapy.

This study has several limitations that should be considered when interpreting our findings. First, the modest sample size (*n* = 15) restricts statistical power to draw definitive clinical conclusions; however, this pilot study serves as a valuable hypothesis-generating framework for future, larger multi-center investigations. Second, the observational nature of the study, while reflecting “real-world” clinical practice, resulted in heterogeneity regarding prior antibiotic exposure. Although we accounted for this in our analysis, the temporal variability in treatment administration remains a confounding factor. Third, the lack of a non-CAP control group prevents us from definitively determining whether the observed microbial profiles—particularly the enrichment of *Ralstonia*—are specific to the disease state or representative of the lower respiratory tract in the local environment. Finally, while 16S rRNA gene sequencing provides a high-resolution view of the bacterial community, it does not distinguish between viable and non-viable organisms, nor does it characterize the fungal or viral components of the pulmonary microbiome. In addition, sequencing the hypervariable region V3–V4 of the 16S rRNA gene may not reliably distinguish closely related species.

Our findings illustrate that 16S rRNA gene sequencing detects bacterial taxa beyond those identified by conventional microbiological methods, although the clinical relevance of these additional detections remains to be established. In this context, sequencing should be considered complementary to, rather than a replacement for, conventional microbiological diagnosis. This contributes to diagnostic uncertainty and may lead to empirical treatment with broad-spectrum antibiotics, exacerbating concerns regarding antimicrobial resistance. The observed differences after antibiotic exposure further emphasize the need for early and accurate microbiological diagnosis to inform antimicrobial selection. These findings may have important clinical implications and open new avenues for research into the role of the lung microbiota in respiratory diseases and the development of rapid diagnostic tools based on novel etiological agents.

## Data Availability

The datasets presented in this study can be found in online repositories. The names of the repository/repositories and accession number(s) can be found in the article/Supplementary material.
